# PATIENT PERCEPTIONS OF USING VOICE-BASED DIETARY ASSESSMENT TOOLS AMONG OLDER ADULTS

**DOI:** 10.1093/geroni/igac059.1475

**Published:** 2022-12-20

**Authors:** Tiffany Driesse, Xiaohui Liang, Michael Fowler, Jing Yuan, Hillary Spangler, David Lynch, John Batsis

**Affiliations:** UNC School of Medicine, University of North Carolina at Chapel Hill, Chapel Hill, North Carolina, United States; University of Massachusetts Boston, Boston, Massachusetts, United States; University of North Carolina at Chapel Hill, Chapel Hill, North Carolina, United States; University of Massachusetts Boston, Boston, Massachusetts, United States; University of North Carolina at Chapel Hill, Chapel Hill, North Carolina, United States; UNC, Chapel Hill, North Carolina, United States; University of North Carolina at Chapel Hill, Chapel Hill, North Carolina, United States

## Abstract

Poor diet among older adults is a risk factor for developing multiple chronic diseases. Dietary recall comprises an important component in intervention research and clinical care. Commonly used tools include the web-based automated self-administered 24-hour assessment (ASA-24). Yet voice assistant (VAS) systems (i.e., Amazon Alexa) have not been developed for this purpose. Hence, we evaluated patient perceptions on performing a VAS-based dietary assessment among older adults. Community-dwelling adults (age 65+ years) participated in two virtual sessions who reported their past 24-hour intake, first using ASA-24, and then using a VAS. All completed a Likert questionnaire (binary, % strongly agree/strongly disagree reported) regarding the simplicity of using both systems, completion time, and user satisfaction. Semi-structured interviews allowed us to ask about technology use. Of the 40 participants (100% enrolled), mean age was 69±1.0 years (85% female, 100% white, 5% Latinx). Only 40% owned a VAS; 60% reported having VAS experience prior to the study. After completing both sessions, 80% preferred a VAS over the ASA-24. Participants reported that web-based recalls were unnecessarily complex (60%), time-consuming (50%), and 60% did not wish to use them. Comparatively, VAS recalls were intuitive (75%), easily reportable (85%), and there was willingness to report food while preparing meals (85%). In 16 participants, we evaluated themes of VAS use including easier navigation, less time, and ability to have a natural conversation. A VAS provides a more convenient, conversational, and computerless interaction to report meals over web-based solutions suggesting they hold promise for dietary recall in older adults.

